# The Intestinal Archaea *Methanosphaera stadtmanae* and *Methanobrevibacter smithii* Activate Human Dendritic Cells

**DOI:** 10.1371/journal.pone.0099411

**Published:** 2014-06-10

**Authors:** Corinna Bang, Katrin Weidenbach, Thomas Gutsmann, Holger Heine, Ruth A. Schmitz

**Affiliations:** 1 Institute for General Microbiology, Christian-Albrechts-University Kiel, Kiel, Germany; 2 Division of Biophysics, Research Center Borstel, Borstel, Germany; 3 Division of Innate Immunity, Research Center Borstel, Airway Research Center North, Member of the German Center for Lung Research (DZL), Borstel, Germany; Institut Pasteur de Lille, France

## Abstract

The methanoarchaea *Methanosphaera stadtmanae* and *Methanobrevibacter smithii* are known to be part of the indigenous human gut microbiota. Although the immunomodulatory effects of bacterial gut commensals have been studied extensively in the last decade, the impact of methanoarchaea in human's health and disease was rarely examined. Consequently, we studied and report here on the effects of *M. stadtmanae* and *M. smithii* on human immune cells. Whereas exposure to *M. stadtmanae* leads to substantial release of proinflammatory cytokines in monocyte-derived dendritic cells (moDCs), only weak activation was detected after incubation with *M. smithii*. Phagocytosis of *M. stadtmanae* by moDCs was demonstrated by confocal microscopy as well as transmission electronic microscopy (TEM) and shown to be crucial for cellular activation by using specific inhibitors. Both strains, albeit to different extents, initiate a maturation program in moDCs as revealed by up-regulation of the cell-surface receptors CD86 and CD197 suggesting additional activation of adaptive immune responses. Furthermore, *M. stadtmanae* and *M. smithii* were capable to alter the gene expression of antimicrobial peptides in moDCs to different extents.

Taken together, our findings strongly argue that the archaeal gut inhabitants *M. stadtmanae* and *M. smithii* are specifically recognized by the human innate immune system. Moreover, both strains are capable of inducing an inflammatory cytokine response to different extents arguing that they might have diverse immunomodulatory functions. In conclusion, we propose that the impact of intestinal methanoarchaea on pathological conditions involving the gut microbiota has been underestimated until now.

## Introduction

Archaea belong to the second domain of Prokarya and their phylogenetic distance to Bacteria and Eukarya is reflected by genetic as well as structural differences [1]. Members of the domain archaea are ubiquitous and exist in a broad variety of habitats ranging from environments with temperatures above 100°C or with very high salinity (extremophiles) to ecosystems with mild growth conditions such as sewages, the oceans and soils (mesophils) [2]–[4]. Since archaea were originally described to occur only in extreme environments, their potential impact in the ecosystem of eukaryotes regarding physiology or pathogenicity was not considered for many years [5]. However, members of the archaea have currently been shown to appear frequently and in high numbers as part of the commensal microbiota found in insects, and mammals including humans [6]–[13]. Particularly, the methanoarchaeon *Methanobrevibacter smithii* has been shown to be the most abundant archaeon within the human intestine comprising up to 10% of all the present anaerobically growing microorganisms, and its quantities within the human gut have been shown to be stable over life time [6], [7], [10], [14]. By converting bacterial primary and secondary fermentation products, like hydrogen and carbon dioxide to methane, *M. smithii* is essential for syntrophic metabolisms within the microbial community in the human gut [7], [15], [16] and thus, plays a crucial role in energy balance [17]. Besides *M. smithii*, an additional member of the Methanobacteriales, *Methanosphaera stadtmanae*, has been detected in human stool samples, although in significant lower abundance [18], [19].

As a result of several reports on the apparent correlation between the quantities of methanoarchaea in the large intestine and the development of severe colon diseases, their role in pathogenicity has been addressed in a small number of studies [20]–[23]. In this respect, only few studies on the microbial diversity in individuals suffering from inflammatory bowel diseases proposed the involvement of human mucosa-associated methanoarchaea [24]. In general, it is currently assumed that methanoarchaea are able to promote the growth of pathogenic microbes and are thus indirectly involved in pathogenicity [5]. On the contrary, Blais-Lecours *et al.* provided evidence for immunogenical properties of *M. smithii* and *M. stadtmanae* in immunized mice and human serum [25], [26]; leading to the conclusion that methanoarchaea might be recognized by innate immune cells. Those cells recognize non-self molecules by numerous membrane-bound, cytosolic or secreted receptors (pattern recognition receptors, PRRs) and are thus the first line of defense against bacteria, fungi or viruses [27], [28]. By recognition and binding of microbe-associated molecular patterns (MAMPs) on the surface of microorganisms they activate innate immune responses such as production of cytokines, activation of the complement cascade or the release of antimicrobial peptides (AMPs) [27]–[29]. However, to date neither archaeal-associated molecular patterns nor human PRRs that recognize archaeal cells have been detected. Taking the unique composition and biochemical structure of methanoarchaeal cell envelopes and membranes as well as the previously detected immunogenic effects of methanoarchaeal cells into account [25], [26], [30]–[32], one can assume that methanoarchaea are (specifically) recognized by human immune cells. Consequently, we aimed to elucidate the activation of human immune cells in response to *M. stadtmanae* or *M. smithii*.

## Materials and Methods

### Ethics statement

Approval for these studies was obtained from the Institutional Ethics Committee at the University of Lübeck (Lübeck, Germany; Az. 12-202A) according to the Declaration of Helsinki. All donors gave written informed consent.

### Strains and media

*M. stadtmanae* (DSM 3091) and *M. smithii* (DSM 861) were grown as described earlier and cell numbers were determined by microscopic counting using a Thoma counting chamber during the growth period [33]. Initial experiments were performed with viable and heat-inactivated (95°C for 10 min) cells of *M. stadtmanae* and *M. smithii* in minimal medium under strict anaerobic conditions at 37°C and in a humidified atmosphere of 5% carbon dioxide at 37°C for testing various viability conditions. Final immune cell stimulation experiments were carried out with exponentially growing *M. stadtmanae* and *M. smithii* cells that were centrifuged at 3200× g for 30 min, washed and resuspended in aerobic 50 mM Tris-HCl (pH 7.0).

### Cell culture

Preparation of moDCs was performed by harvesting peripheral blood mononuclear cells (PBMCs) from heparinized blood of donors by Ficoll (PAA Laboratories GmbH, Pasching, Austria) separation [34] and subsequent isolation of monocytes by counter flow elutriation centrifugation [35]. MoDCs were then generated as described previously [36], harvested and re-cultured in RPMI medium (PAA Laboratories GmbH) supplemented with 10% FCS (Biochrom AG), 2 mmol/L glutamine (PAA Laboratories GmbH) and antibiotics (100 U/ml penicillin and 100 µg/ml streptomycin (both PAA Laboratories GmbH)) for stimulation experiments.

Caco-2/BBe cells (ATCC # CRL-2102, a gift from Dr. A. Frey, Research Center Borstel) were grown in DMEM with high glucose and stable L-glutamine (PAA Laboratories GmbH, Pasching, Austria) supplemented with 10% FCS (Biochrom AG, Berlin, Germany), 1% penicillin/streptomycin (PAA Laboratories GmbH), 10 µg/ml human transferrin (Roche Deutschland Holding GmbH) and 1 mM sodium pyruvate (PAA Laboratories GmbH).

Growth and transfection of HEK293 cells with the respective expression plasmids was carried out as described previously [37], [38] with the following additions: FLAG-TLR3 and -TLR5 have been obtained from P. Nelson (Seattle, USA); TLR7, 8, and 9 have been obtained from B. Beutler (Dallas, USA) and subcloned into pcDNA3.1 (Life Technologies GmbH). The respective control stimuli Poly IC, R848, Flagellin, *E. coli* K12, DAP and MDP were obtained from InvivoGen (San Diego, USA), Pam_3_CSK_4_ was purchased from EMC microcollections GmbH (Tübingen, Germany) and LPS from *Salmonella enterica* sv. Friedenau was a gift from Dr. Helmut Brade (Research Center Borstel).

All cells were grown and incubated in a humidified atmosphere of 5% carbon dioxide at 37°C.

### Cytokine Measurements

Released cytokine concentrations in supernatants of moDCs after 24 h were determined by using commercial ELISA Kits (Life Technologies GmbH) specific for IL-1β, IL-8 and TNF-α.

### Confocal laser scanning microscopy

For confocal laser scanning microscopy 10^5^ moDCs were incubated at 37°C for 2 h on glass cover slips prior addition of 1 µM CytD in DMSO or DMSO alone for control. After 30 min preincubation, moDCs were stimulated with methanoarchaeal cells for 4 h and labeled with LysoTracker Red DND-99 (1∶1000, Sigma-Aldrich Chemie GmbH, Hamburg, Germany). After fixation in 3% paraformaldehyde, moDCs were labeled with Hoechst 33342 (1∶3000, (Life Technologies GmbH)). Images were captured using LSM 510 confocal microscopy (Zeiss) with Leica confocal software.

### Electron microscopy

For TEM analysis 10^6^ moDCs were stimulated for 4 h with 10^8^ methanoarchaeal cells in 24-well plates (Corning Incorporated), washed with PBS and resuspended in 1 ml PBS containing 2.5% glutaraldehyde. The following probe preparation and TEM analysis was performed as described previously [39].

### Quantitative Real-Time (RT)PCR

Total RNA was isolated by the use of NucleoSpin RNA II kit (Macherey-Nagel GmbH & Co. KG, Düren, Germany) and reverse-transcribed using the SuperScript III Reverse Transcriptase (Life Technologies GmbH). Primers that were used for quantitative RT-PCR analysis are listed in Table 1. Amplification was carried out in a LightCycler 480 instrument (Roche Deutschland Holding GmbH, Grenzach-Wyhlen, Germany) using the SYBR Green I Mastermix (Roche Deutschland Holding GmbH) according to the manufacturer's protocol. Relative quantification of mRNA was calculated with the LightCycler 480 Software, Version 1.5.

**Table 1:** Primer used for quantitative RT-PCR.

Primer name	Forward primer (5′→3′)	Reverse primer (3′→5′)
LL37/CAMP18	TCGGATGCTAACCTCTACCG	ACAGGCTTTGGCGTGTCT
HBD1	TGTCTGAGATGGCCTCAGGT	GGGCAGGCAGAATAGAGACA
HBD2	TCAGCCATGAGGGTCTTGTA	GGATCGCCTATACCACCAAA
HBD3	TTCTGTTTGCTTTGCTCTTCC	CGCCTCTGACTCTGCAATAA
HBD4	GCCGGAAGAAATGTCGCAGC	AGCGACTCTAGGGACCAGCA
HD5	GCCATCCTTGCTGCCATTC	TGATTTCACACACCCCGGAGA
HD6	CCTCACCATCCTCACTGCTGTTC	CCATGACAGTGCAGGTCCCATA
HPRT	GTCAGGCAGTATAATCCAAAGA	GGGCATATCCTACAACAAACT
TNF-α	CCTGTAGCCCATGTTGTAGCA	TTGAAGAGGACCTGGGAGTAG
IL-1β	TGGGCCTCAAGGAAAAGAATC	GGGAACTGGGCAGACTCAAAT
IL-8	TTGCCAAGGAGTGCTAAAGAA	CAACCCTACAACAGACCCACAC

### Fluorescence activated cell sorting (FACS)

FACS analysis of 2×10^5^ moDCs after 24 and 48 h of stimulation with the methanoarchaeal strains was performed after washing moDCs with phosphate buffered saline (PBS) containing sodium azide, centrifugation and incubation for 30 min with antibodies labeled either with phycoerythrin (PE) or fluorescein isothiocyanate (FITC) (BD Biosciences, San Rose, USA). Labeled moDCs were washed, fixed in PBS and 3% paraformaldehyde and analyzed with a FACS flow cytometer (LSRII, BD Biosciences) by using BD FACSDiva Version 6. MoDCs were selected by forward and side scattered signals before measuring the intensity of PE or FITC fluorescence signals of 10000 cells. Shown graphics were performed with FlowJo Software, Version 7.5.5.

### Statistical analysis

Statistics were performed with GraphPad Prism 5.02 software (GraphPad, San Diego, CA, USA) with differences *p<0.05, **p<0.01 and ***p<0.001 considered significant.

## Results and Discussion

### Immune reaction of intestinal epithelial cells in response to *M. stadtmanae*- and *M. smithii*-stimulation

Since *M. stadtmanae* and *M. smithii* were found to be inhabitants of the human gut, we initially examined cell activation of the intestinal epithelial cell line Caco-2/BBe concerning expression and release of different proinflammatory cytokines and several AMPs. However, neither cytokine release of IL-8 nor significant changes in transcript levels of genes encoding TNF-α, IL-8, human beta defensin 1 (HBD1), HBD4, human defensin 6 (HD6) or human cathelicidin LL37 after stimulation with *M. stadtmanae* or *M. smithii* were observed (Fig. S1). These findings strongly argue that *M. stadtmanae* and *M. smithii* are not recognized by human intestinal epithelial cells. Taking this observation into account and the fact that innate immune cells get in contact with epithelial invading microorganisms from the human gut, the following experiments were performed with human monocyte-derived dendritic cells (moDCs).

### Activation of monocyte-derived dendritic cells in response to *M. stadtmanae* and *M. smithii*

**Figure 1:**
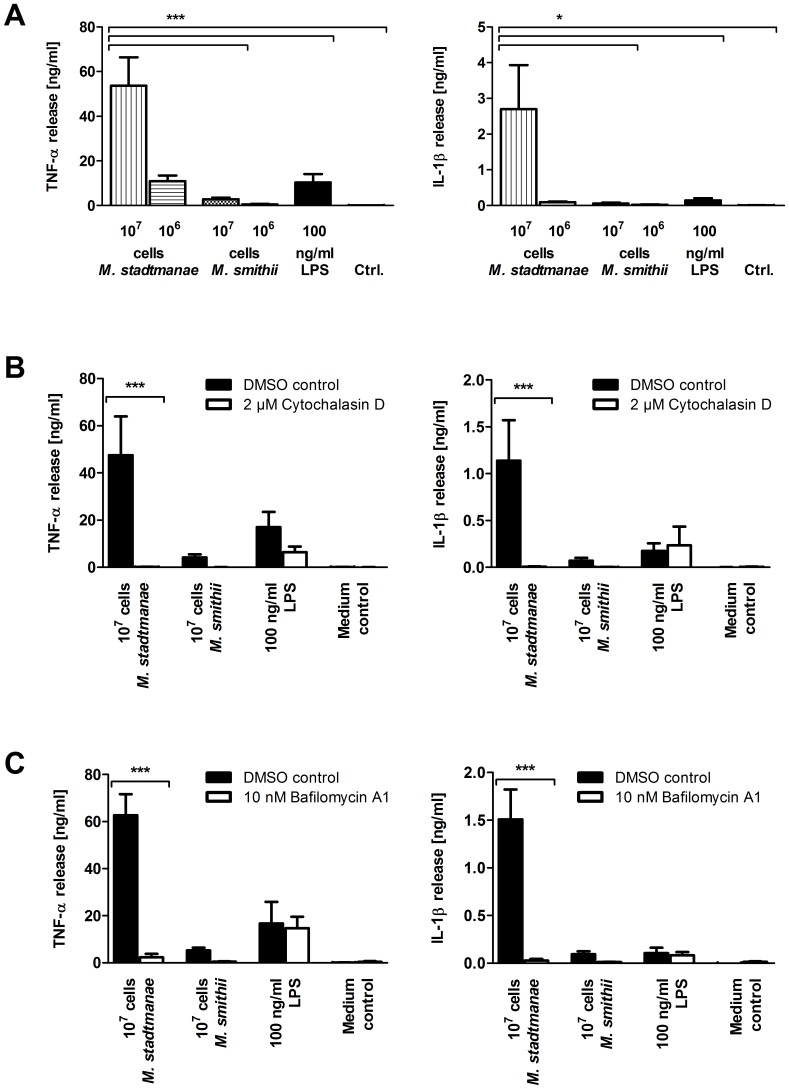
*M. stadtmanae* induces significant higher release of cytokines in moDCs than *M. smithii*. Cytokine release after stimulation of 2×10^5^ moDCs with 1×10^6^ and 1×10^7^ *M. stadtmanae* or *M. smithii* cells for 20 h was quantified using commercial ELISA-Kits (**A**–**C**). MoDCs were preincubated with 2 µM Cytochalasin D in DMSO (**B**) or 10 nM Bafilomycin A1 in DMSO (**C**) for 30 min prior stimulation. LPS (100 ng/ml) and medium were used as controls. Stated data are means of at least 3 independent biological replicates with their respective standard errors of the mean (SEM).

Activation of 2×10^5^ moDCs from at least three donors was evaluated by stimulation with 10^6^ and 10^7^ *M. stadtmanae* or *M. smithii* cells for 20 h and subsequent analysis of TNF-α and IL-1β release. High amounts of both cytokines monitored were detected after stimulation with *M. stadtmanae* in a cell concentration-dependent manner, whereas *M. smithii* in general lead to a comparably weak release of the tested cytokines (Fig. 1A). This finding of a stronger immune cell activation by *M. stadtmanae* is in agreement with previous observations of Blais-Lecours *et al.* [25]; revealing much higher accumulation of myeloid dendritic cells and higher induction of antigen-specific IgGs in plasma of mice and human after intranasal application of *M. stadtmanae* cells when compared to *M. smithii*. In addition, it has very recently been demonstrated that lyophilized cells of *M. stadtmanae* induce significant higher release of TNF-α by peripheral blood mononuclear cells (PBMCs) compared to *M. smithii* [26].

### Phagocytosis of *M. stadtmanae* and *M. smithii* is crucial for activation of monocyte-derived dendritic cells

**Figure 2:**
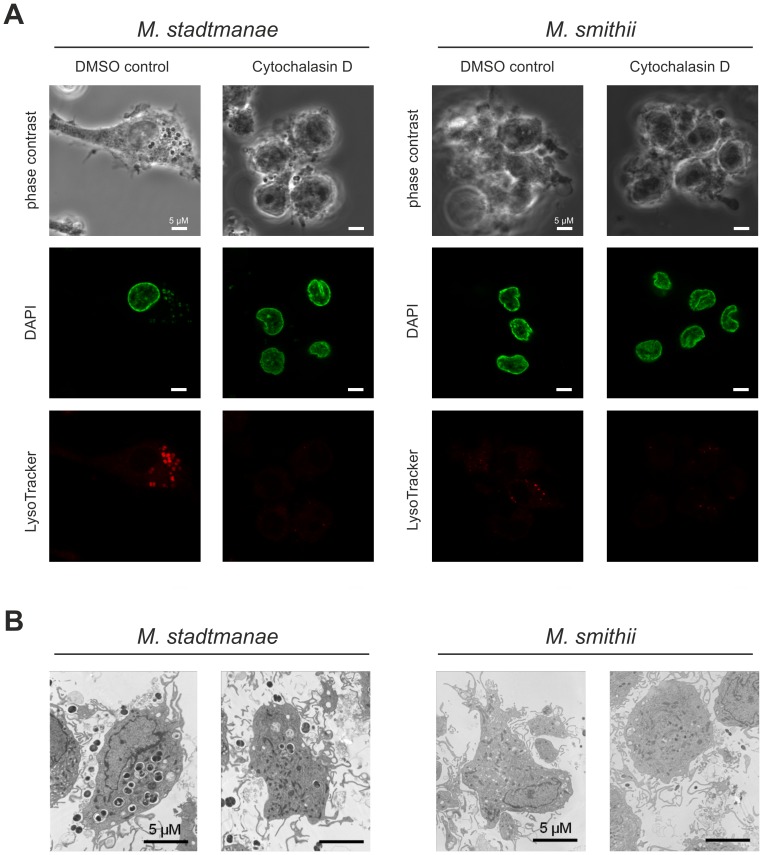
Phagocytosis of *M. stadtmanae* is crucial for immune cell activation. **A)** After preincubation with 1 µM Cytochalasin D (in DMSO) and with DMSO alone (control) for 30 min 1×10^5^ moDCs were stimulated with 1×10^7^ methanoarchaeal cells in glass cover slips for a period of 4 h. Lysosomes in moDCs were stained with LysoTracker Red DND-99 during time of incubation. After incubation, moDCs were washed, fixed with 3% paraformaldehyde and labeled with Hoechst (DAPI-staining). Images were captured using LSM 510 confocal microscopy (Zeiss) with Leica confocal software and are representative of the respective samples. **B)** 1×10^6^ moDCs were stimulated with 1×10^8^ methanoarchaeal cells for a period of 4 h. After washing in PBS, moDCs were fixed for electron microscopy. Images are representative for the respective sample.

Since uptake of living commensal microorganisms by immune cells in the human gut has been shown to be crucial for cell activation by several bacterial species [40], we further investigated whether phagocytosis is involved in cell activation by methanoarchaea. Thus, phagocytosis and the effects of Cytochalasin D (Cyt D) and Bafilomycin A1 (Baf A1) on moDCs was monitored during stimulation with *M. stadtmanae* or *M. smithii*. Cyt D is known to specifically inhibit the uptake of microorganisms, whereas Baf A1 prevents intracellular lysosome formation. The cytokine release by moDCs monitored after stimulation with both methanoarchaeal strains was significantly inhibited upon treatment with Cyt D (Fig. 1B) and Baf A1 (Fig. 1C), whereas LPS-activation (control) was not affected. Furthermore, DAPI-prestained moDCs were visualized using confocal microscopy and revealed rapid phagocytosis of *M. stadtmanae* after 4 h of incubation (Fig. 2A, *M*. *stadtmanae*, Control, DAPI). Prestaining of moDCs with LysoTracker displayed lysosome formation after 4 h of incubation with *M. stadtmanae* (Fig. 2A, *M*. *stadtmanae*, Control, LysoTracker). For verification, moDCs were preincubated with 1 µM Cyt D and subsequently stimulated with *M. stadtmanae*. In this experimental setup, due to the Cyt D treatment *M. stadtmanae* cells were no longer visible inside moDCs (Fig. 2A, *M*. *stadtmanae*, Cytochalasin D, DAPI) and lysosome formation was not detected (Fig. 2A, *M*. *stadtmanae*, Cytochalasin D, LysoTracker). In contrast, stimulation of moDCs with *M. smithii* in the same experimental setup did not reveal uptake or lysosome formation after 4 h of stimulation (Fig. 2A, *M*. *smithii*). Moreover, TEM analysis of moDCs after 4 h of stimulation with *M. stadtmanae* or *M. smithii* confirmed extensive uptake of *M. stadtmanae* cells by moDCs, whereas uptake of *M. smithii* was not detected (Fig. 2B). These findings strongly indicate that *M. stadtmanae* cells are rapidly phagocytosed by human immune cells and, moreover, this uptake is crucially required for cellular activation. In contrast to *M. stadtmanae*, phagocytosis of *M. smithii* by moDCs appeared to be less frequent or much slower; nevertheless, cytokine release appeared as well to be dependent on phagocytosis.

### Activation and modulation of moDCs by *M. stadtmanae* and *M. smithii*

**Figure 3:**
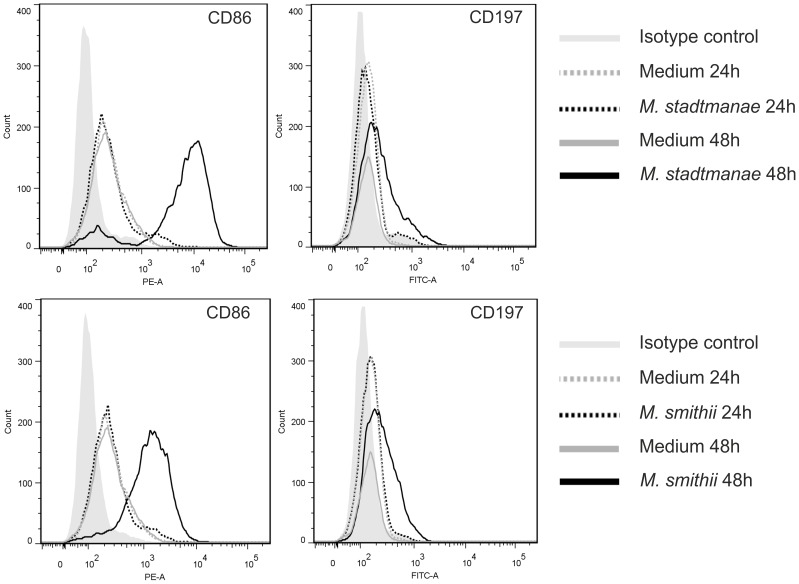
Increased expressions of cell-surface receptors on moDCs after stimulation with *M. stadtmanae* and *M. smithii*. 1×10^6^ moDCs were stimulated with 1×10^7^ *M. stadtmanae* or *M. smithii* cells or medium for 24 h and 48 h at 37°C. Subsequently, 2×10^5^ moDCs were incubated with antibodies (PE- or FITC-labeled) and analyzed with a FACS flow cytometer. MoDCs were selected by forward and side scattered signals before measuring the intensity of PE or FITC fluorescence signals of 10000 cells. Depicted graphics are original plots of FlowJo Software, Version 7.5.5.

DCs are known to act as crucial messengers between innate and adaptive immunity. In particular, activated moDCs maturate and migrate from nonlymphoid tissues to lymphoid organs to initiate T cell-mediated immune responses [27], [41]. Thus, the cell-surface expressions of CD197 and of the co-stimulatory receptor CD86 were investigated. MoDCs were stimulated with *M. stadtmanae* and *M. smithii* or medium as control for 24 and 48 h followed by incubation with the respective antibodies directed against CD86 and CD197. The subsequent FACS analyses of these cells revealed increased expression of both cell-surface receptors on moDCs after stimulation with both, *M. stadtmanae* and *M. smithii*, whereas medium controls were not affected (Fig. 3). The expression of both cell-surface receptors on moDCs however, was found to be higher after stimulation with *M. stadtmanae* compared to *M. smithii*. Since the expression of CD86 and CD197 after activation is crucial for co-stimulatory signals that are involved in maturation of moDCs and their functions in adaptive immune responses such as T- and B-cell activation [41], [42], those results implicate activation not only of the innate but also of the adaptive immune system in response to *M. stadtmanae* and *M. smithii*. This assumption is further supported by a very recently published study, demonstrating development of a significant and specific anti-*M. stadtmanae* IgG response in patients suffering from inflammatory bowel diseases (IBD) [26].

**Figure 4:**
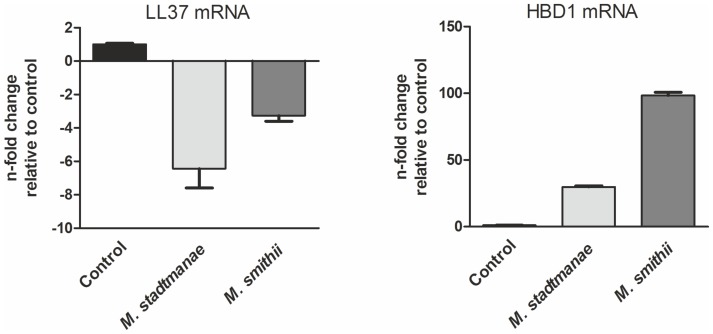
Altered gene expression of AMPs in moDCs after stimulation with *M. stadtmanae* and *M. smithii*. 2–4×10^6^ moDCs were stimulated with 1×10^7^ *M. stadtmanae* or *M. smithii* cells and medium (for control) over a period of 24 h. Subsequently, RNA was isolated according to manufacturer's protocol and reverse-transcribed. Relative quantification of expression of genes encoding HBD1 and LL37 were calculated with the LightCycler 480 Software in relation to house-keeping gene *hprt*.

We further aimed to determine the expression of various human antimicrobial peptides in stimulated moDCs by qRT-PCR. Thus, moDCs were stimulated for 24 h with the methanoarchaeal strains prior isolating their respective RNA and quantifying gene expression of genes encoding AMPs. In this respect, HBD1 gene expression was found to be up-regulated in moDCs in response to both, *M. stadtmanae* and *M. smithii* (Fig. 4). Gene expression of further antimicrobial peptides in moDCs such as HBD2, HBD3 and RNase7, was not detectable using qRT-PCR analysis. DCs are clearly not major producers of the antimicrobial peptide response, however HBD1 has been shown to be selectively chemotactic for human intestinal DCs [43]. Thus, the regulation of HBD1 gene expression in moDCs in response to stimulation with *M. stadtmanae* and *M. smithii* might link our results to the physiological functions of those methanoarchaea as immunomodulators in the human gut.

Besides, we found that the expression level of the human cathelicidin LL-37 was down-regulated in moDCs after stimulation with *M. stadtmanae* (6-fold) and *M. smithii* (3-fold). The regulation of LL37 by different bacterial components in various disease patterns has been observed in earlier studies [44]. In enteric infections, for example, it was proposed that bacterial DNA actively down-regulates the gene expression of LL37 in monocytes and epithelial cells [45]. However, since methanoarchaea are considered to be commensals within the human intestine, it might also thinkable that they evolved mechanisms protecting themselves from human immune clearance. This would be in accordance with our recently published data on the susceptibility of methanoarchaea against numerous AMPs, in particular against LL32 [33] that is described as the shortest active unit of human LL37 [46].

### Recognition of *M. stadtmanae* and *M. smithii* by human immune cells

Based on the observed rapid activation processes of moDCs after stimulation with *M. stadtmanae* shown by confocal scanner microscopy analyses during this study, we propose a specific recognition mechanism for *M. stadtmanae*. This mechanism might differ from that of *M. smithii*. The cell envelope of both, *M. stadtmanae* and *M. smithii*, is in general built up by a dense layer of pseudomurein, which consists of glycan strands consisting of β-1,3-glycosidic linked *N*-acetylglucosamine and *N*-acetyltalosaminuronic acid, and a variable peptide moiety [47]–[49]. However, structural alterations of pseudomurein in the cell envelope of *M. stadtmanae* and *M. smithii* might be responsible for the obtained differences in the recognition process by human immune cells, since studies employing monoclonal antibodies against methanoarchaea by Conway de Macario and colleges revealed diverse immunogenic properties by several pseudomurein glycan structures of *M. smithii* fecal isolates [30], [31], [50]. Genomic heterogeneity of *M. smithii* populations present in the gut microbiota of individuals has already been described earlier [51]. Thus, alterations of the methanoarchaeal cell envelope might occur in case of *M. smithii* isolates derived from diverse human individuals that may also result in diverse immunogenic properties of these strains. Although the overall structure of pseudomurein in some parts resembles that of murein [49], we obtained evidence that *M. stadtmanae* or *M. smithii* cells are not recognized by human NOD1- and 2 receptors, which are known to be activated by bacterial murein components (Fig. S2). Moreover, by transfection of common TLRs into HEK293-cells we also get strong indication that none of the so far known members of the human toll-like receptor family [52] appears to be involved in the recognition processes of *M. stadtmanae* or *M. smithii* cells (Fig. S2). Hence, activation of immune cells by *M. stadtmanae* and *M. smithii* appears not to occur via commonly known TLRs or NLRs that recognize prominent bacterial MAMPs, strongly pointing towards a different recognition mechanism.

### The functional role of *M. stadtmanae* and *M. smithii* in the human intestine

The existence of methanoarchaea as a part of the human gut microbiota has been accepted in the last two decades [6], [7], [19], [24], however their impact on the immune system in human's health and disease was hardly examined. Even today, they are still overlooked in many studies dealing with the interdependency between members of the microbiome and components of the immune system. Due to the broad variety of detection assays the abundance and diversity of archaea in the human gut is still not fully elucidated and remains indistinct [10], [24]. Hence, the current knowledge on the functional role of methanoarchaea in the human intestine is mainly focused on bioenergetic aspects and syntrophic interactions with bacteria [12], [16]. However, few studies reported strong immunological properties of methanoarchaea after immunization of rabbits and mice [25], [26], [30], [31], [50]. Thus, it is most likely that methanoarchaea are also capable to influence the community structure of the human gut microbiota through their interaction with blood immune cells or the mucosa itself. Remarkably, by using an adapted DNA-isolation and qRT-PCR approach, Dridi *et al.* demonstrated that *M. smithii* inhabits nearly every human individual gut ecosystem, whereas *M. stadtmanae* is less abundant (29%) [19]. Moreover, *M. stadtmanae* was recently found to be more abundant in patients suffering from IBD than in healthy control individuals [26]. Taking those findings and our results on *M. stadtmanae*'s severe activation of moDCs into consideration, it appears that the presence of *M. stadtmanae* might directly or indirectly correlate with inflammation processes in the human gut.

Regarding the overall immunogenic potential of methanoarchaeal strains this study focuses on the strains *M. stadtmanae* (DSM 3091) and *M. smithii* (DSM 861), however other isolates of these strains as well as further methanoarchaeal strains inhabiting the human intestine such as *Methanomassilicoccus luminyensis* [53] might elicit far diverse immune responses when exposed to human epithelial or blood immune cells.

### Conclusions

We report here on the inflammatory response of human moDCs to methanoarchaea and demonstrate that *M. stadtmanae* is capable to induce a markedly higher inflammatory cytokine response than *M. smithii*, and may represent a hitherto overlooked contributor to pathological conditions in the human intestine. Moreover, our data implicate the presence of a specific archaeal-associated pattern recognition receptor in humans. Since members of the domain Archaea were not only found in the human intestine, but also in the oral cavity and in high abundance on human skin [6]–[12], [19], archaeal strains may influence the overall human immune homeostasis to comparable extents as has been shown for bacteria. Consequently, there is an urgent need to include archaea in future studies regarding the role of the human microbiome.

## Supporting Information

Figure S1 **Stimulation of intestinal epithelial cells does not reveal activation by** ***M. stadtmanae*** **and** ***M. smithii*** **. A)** Cytokine release after stimulation of 1×10^5^ Caco-2/BBe cells with 1×10^7^ *M. stadtmanae* or *M. smithii* cells for 20 h was quantified using commercial ELISA-Kits. TNF-α (10 ng/ml), medium and 1×10^7^ *E. coli* K12 were used as controls. Stated data are means of 3 independent biological replicates with their respective standard errors of the mean (SEM). **B)** 1×10^6^ Caco-2/BBe cells were stimulated with 1×10^7^ *M. stadtmanae* or *M. smithii* cells, TNF-α (10 ng/ml), medium and 1×10^7^ *E. coli* K12 cells (for control) over periods of 6 h and 24 h. After RNA isolation (Macherey-Nagel) and reverse transcription the relative quantification of TNF-α, IL-8, HBD1, HD6, HBD4 and LL37 mRNA expression was carried out in relation to house-keeping gene *hprt* calculated with the LightCycler 480 Software. Stated data are means of 3 independent biological replicates with their respective SEM.
(PDF)

Figure S2 **Transfection of HEK293 cells with common TLRs or NLRs does not reveal activation by** ***M. stadtmanae*** **or** ***M. smithii*** **via these receptors.** 2.5×10^4^ HEK cells were transfected with various common human innate immune receptors as indicated. Cells were then stimulated with 1×10^7^ *M. stadtmanae* and *M. smithii* cells or the indicated control stimuli (Pam3CSK4, Pam3Cys-Ser-(Lys)4; Poly IC, polyriboinosinic:polyribocytidylic acid; LPS, lipopolysachharide; R848, resiquimod; DAP, *diaminopimelic acid*; MDP, *muramyl dipeptide*) for 20 h and IL-8 release was quantified using a commercial ELISA-Kit. Stated data are means of at least 3 independent biological replicates with their respective SEM.
(PDF)
