# Supplementary material for: The Intestinal Archaea Methanosphaera stadtmanae and Methanobrevibacter smithii Activate Human Dendritic Cells
Source: PLoS One. 2014 Jun 10;9(6):e99411. doi: 10.1371/journal.pone.0099411 (PMC4051749; doi:10.1371/journal.pone.0099411)
Supplement: Figure S1 — Stimulation of intestinal epithelial cells does not reveal activation by M. stadtmanae and M. smithii . A) Cytokine release after stimulation of 1×10^5^ Caco-2/BBe cells with 1×10^7^ M. stadtmanae or M. smithii cells for 20 h was quantified using commercial ELISA-Kits. TNF-α (10 ng/ml), medium and 1×10^7^ E. coli K12 were used as controls. Stated data are means of 3 independent biological replicates with their respective standard errors of the mean (SEM). B) 1×10^6^ Caco-2/BBe cells were stimulated with 1×10^7^ M. stadtmanae or M. smithii cells, TNF-α (10 ng/ml), medium and 1×10^7^ E. coli K12 cells (for control) over periods of 6 h and 24 h. After RNA isolation (Macherey-Nagel) and reverse transcription the relative quantification of TNF-α, IL-8, HBD1, HD6, HBD4 and LL37 mRNA expression was carried out in relation to house-keeping gene hprt calculated with the LightCycler 480 Software. Stated data are means of 3 independent biological replicates with their respective SEM. (PDF) [file pone.0099411.s001.pdf]

Figure SI 1

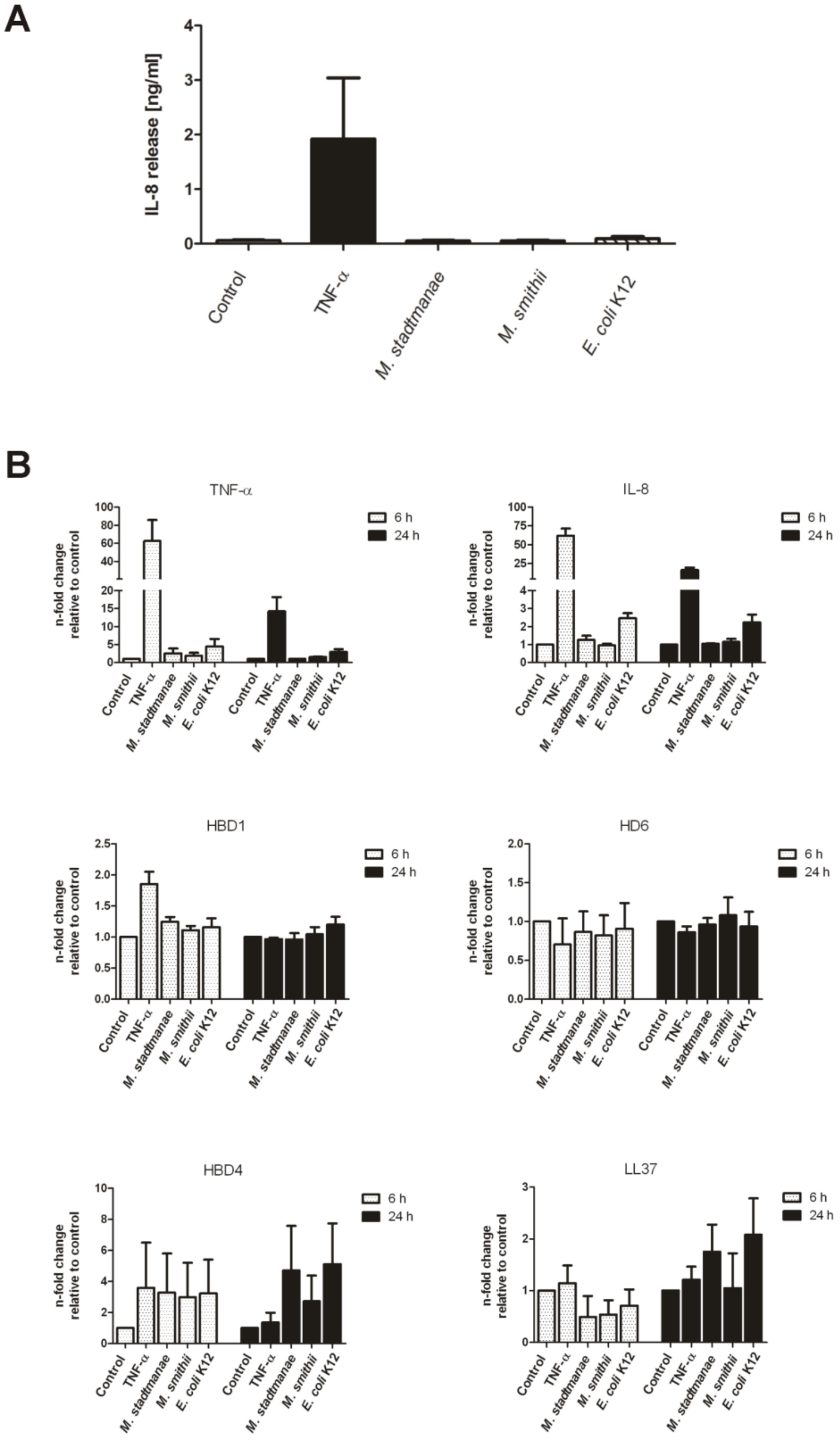
A
IL-8 release [ng/ml]
4
3
2
1
0
Control
TNF-α
M. stadtmanae
M. smithii
E. coli K12
B
TNF-α
IL-8
HBD1
HD6
HBD4
LL37
n-fold change relative to control
6 h
24 h
100
80
60
40
20
15
10
5
0
75
50
25
4
3
2
2.5
2.0
1.5
1.0
0.5
0.0
8
6

Figure SI 1 Stimulation of intestinal epithelial cells does not reveal activation by *M. stadtmanae* and *M. smithii*

A) Cytokine release after stimulation of 1×10^5 Caco-2/BBe cells with 1×10^7 *M. stadtmanae* or *M. smithii* cells for 20 h was quantified using commercial ELISA-Kits. TNF-α (10 ng/ml), medium and 1×10^7 *E. coli* K12 were used as controls. Stated data are means of 3 independent biological replicates with their respective standard errors of the mean (SEM). **B)** 1×10^6 Caco-2/BBe cells were stimulated with 1×10^7 *M. stadtmanae* or *M. smithii* cells, TNF-α (10 ng/ml), medium and 1×10^7 *E. coli* K12 cells (for control) over periods of 6 h and 24 h. After RNA isolation (Macherey-Nagel) and reverse transcription the relative quantification of TNF-α, IL-8, HBD1, HD6, HBD4 and LL37 mRNA expression was carried out in relation to house-keeping gene *hprt* calculated with the LightCycler® 480 Software. Stated data are means of 3 independent biological replicates with their respective SEM.
