# Supplementary material for: The Intestinal Archaea Methanosphaera stadtmanae and Methanobrevibacter smithii Activate Human Dendritic Cells
Source: PLoS One. 2014 Jun 10;9(6):e99411. doi: 10.1371/journal.pone.0099411 (PMC4051749; doi:10.1371/journal.pone.0099411)
Supplement: Figure S2 — Transfection of HEK293 cells with common TLRs or NLRs does not reveal activation by M. stadtmanae or M. smithii via these receptors. 2.5×10^4^ HEK cells were transfected with various common human innate immune receptors as indicated. Cells were then stimulated with 1×10^7^ M. stadtmanae and M. smithii cells or the indicated control stimuli (Pam3CSK4, Pam3Cys-Ser-(Lys)4; Poly IC, polyriboinosinic:polyribocytidylic acid; LPS, lipopolysachharide; R848, resiquimod; DAP, diaminopimelic acid; MDP, muramyl dipeptide) for 20 h and IL-8 release was quantified using a commercial ELISA-Kit. Stated data are means of at least 3 independent biological replicates with their respective SEM. (PDF) [file pone.0099411.s002.pdf]

Figure SI 2

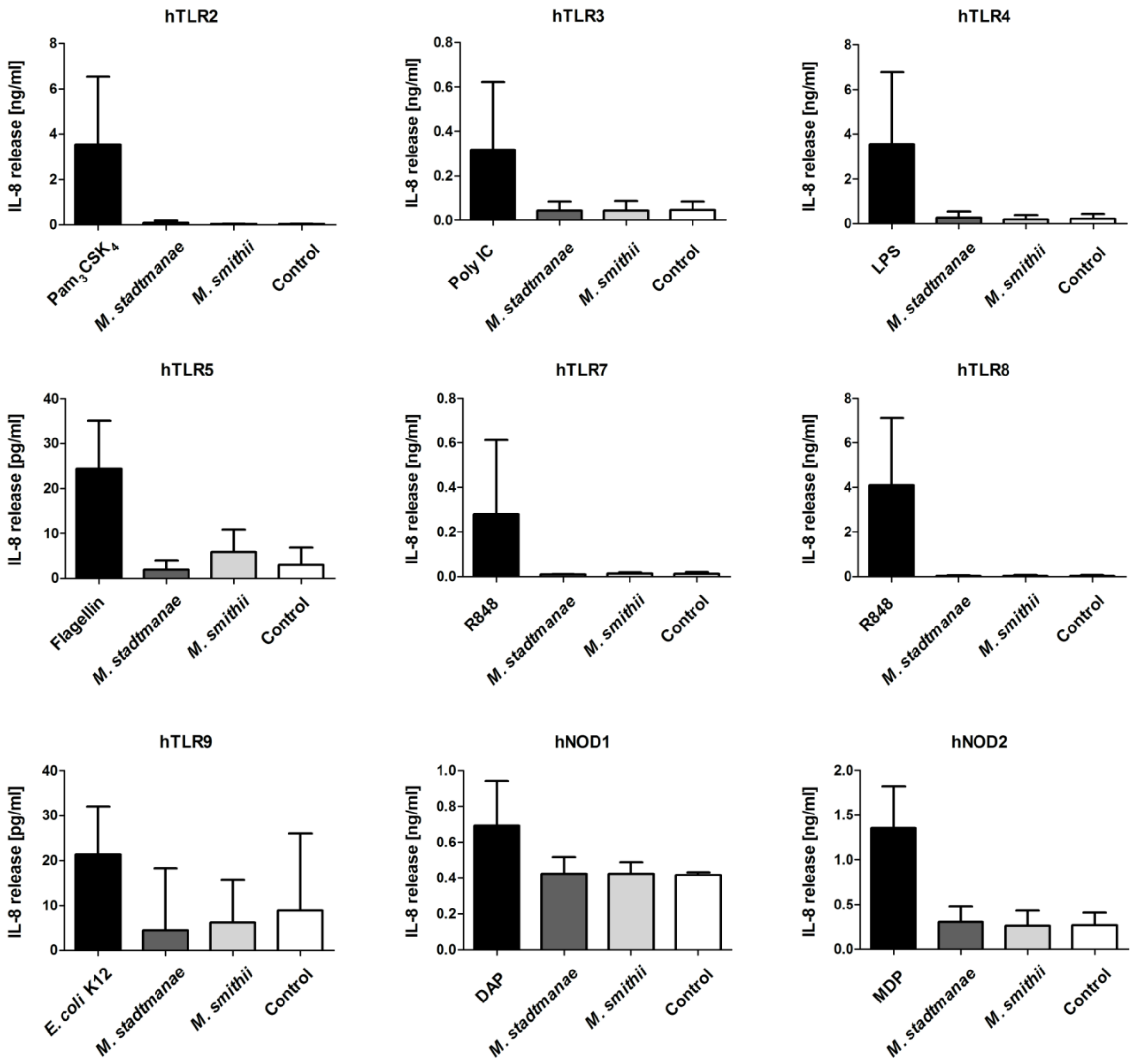

Figure SI 2 Transfection of HEK293 cells with common TLRs or NLRs does not reveal activation by *M. stadtmanae* or *M. smithii* via these receptors

2.5×10^4 HEK cells were transfected with various common human innate immune receptors as indicated. Cells were then stimulated with 1×10^7 *M. stadtmanae* and *M. smithii* cells or the indicated control stimuli (Pam3CSK4, Pam3Cys-Ser-(Lys)4; Poly IC, polyriboinosinic:polyribocytidylic acid; LPS, lipopolysachharide; R848, resiquimod; DAP, *diaminopimelic acid*; MDP, *muramyl dipeptide)* for 20 h and IL-8 release was quantified using a commercial ELISA-Kit. Stated data are means of at least 3 independent biological replicates with their respective SEM.
